# High-risk groups for alpha-gal sensitization 

**DOI:** 10.5414/ALX02424E

**Published:** 2023-08-22

**Authors:** Marie Benders-Guedj, Martin Köberle, Heidelore Hofmann, Tilo Biedermann, Ulf Darsow

**Affiliations:** Department of Dermatology and Allergy Biederstein, Faculty of Medicine, Technical University of Munich, Munich, Germany

**Keywords:** alpha-gal sensitization, borreliosis, tick contact

## Abstract

Background: Tick bite-induced IgE-mediated reactions to the oligosaccharide galactose α-1,3-galactose (alpha-gal) are increasingly recognized. This study investigated alpha-gal sensitization in three groups with different tick bite exposure. Materials and methods: Specific IgE antibodies to alpha-gal and total IgE were investigated in 485 patients with Lyme borreliosis with different disease manifestations and compared to a control group of 200 randomly selected patients without increased exposure to tick bites. A group of 232 hunters and forest workers served as a model for multiple tick bites. Results: Specific IgE (sIgE) antibodies to alpha-gal (> 0.1 kU/L) were found in 12.6% of all borreliosis samples compared to the control group with 9% (relative risk 1.4; 95% CI 0.85 – 2.3; not significant (n.s.). The highest prevalence of sIgE to alpha-gal was observed in hunters and forest service employees (22.8%, relative risk 2.5; 95% CI 1.5 – 4.2; p < 0.001). Higher age and elevated total IgE were also associated with alpha-gal sensitization. Conclusion: IgE sensitization to alpha-gal tends to be more frequent in tick-exposed patients with borreliosis than in controls (n.s.). Moreover, hunters and forest workers show an even higher rate of elevated IgE to alpha-gal. Thus, frequent tick contact may result in alpha-gal sensitization. In the area of Munich, the prevalence of alpha-gal sensitization appears lower than in the state of Baden-Württemberg and lower than in the USA, which may be due to the difference in tick species or the frequency of tick exposure. This study could show that alpha-gal sensitization and presumably alpha-gal syndrome does not seem to be a modern problem but existed already more than 30 years ago.

## Introduction 

Alpha-gal syndrome, a delayed type I red meat allergy based on the production of specific IgE (sIgE) antibodies to the oligosaccharide galactose α-1,3-galactose, is a unique example for allergic reactions to a carbohydrate. Since its description 10 years ago, alpha-gal syndrome has attracted increasing attention due to the severe anaphylactic symptoms and the peculiar origin of individual sensitizations [1, 2, 3, 4]. The known distribution of immediate reactions to cetuximab (which contains alpha-gal moieties) is similar to the areas with Rocky Mountain spotted fever and to areas with frequent exposure to the lone star tick [4, 5, 6]. Subsequently, alpha-gal was stained in the gastrointestinal tract of the castor bean tick (*Ixodes ricinus*) in a Swedish study, which supported the hypothesis on alpha-gal transmission to the host by a tick, followed by sensitization [7, 8]. Patients showed IgE antibodies to the tick species *Ixodes ricinus*, but titers were lower than to alpha-gal [9]. Tick-derived saliva containing residual mammalian glycoproteins, supposedly from mammalian blood meals, is commonly hypothesized to induce an immune response or an IgE class switch [6, 9, 10, 11, 12, 13]. Subcutaneous injection of tick saliva was able to induce antibodies to alpha-gal in a knockout mouse model [14]. Furthermore, allergic reactions often started with itching at the site of a previous tick bite before generalized anaphylaxis followed in sensitized individuals (recall urticaria [15]). Persistent local reactions (itching for two or more weeks) have been shown to represent a typical sign of sensitization, and furthermore indicated that tick bites are involved in the specific antibody production to alpha-gal [2, 3, 6, 8]. 80% of alpha-gal-allergic patients reported tick bites prior to the onset of symptoms [12]. 

When comparing different regions and geographic areas all over Europe, an overall prevalence of sensitization to alpha-gal between 5.5 and 24.5% could be observed, with significantly elevated sensitization rates in rural regions [17, 19]. A strong correlation between alpha-gal-specific IgE, total IgE, and sIgE to *Ixodes ricinus* was described in Sweden. Higher sIgE to alpha-gal was measured in allergic individuals compared to the sensitized borreliosis patients without allergic symptoms [10]. This leads to the hypothesis that apart from exposure aspects, atopy is an important predisposing factor to develop alpha-gal sensitization especially in males and with specific antibody titers to alpha-gal above 0.35 kU/L. Recent studies confirmed a correlation between total IgE levels in atopic individuals and the development of specific IgE antibodies to alpha-gal. As many as 44.4% of alpha-gal-positive individuals were atopic [17], with increased alpha-gal sIgE in patients with previous tick bites during the last year. Repeated tick bites were shown to increase the likelihood for alpha-gal sensitization regardless of the age of the individuals. Tick bites were also shown to be an important predisposing factor for new-onset red meat allergy after a long-held oral tolerance to mammalian meat [18]. As sensitized patients were most commonly over 60 years old [16], a lifetime cumulative factor can be assumed. 

One attempt to improve our understanding of ways to sensitization against alpha-gal is to study populations with single or more frequent tick bites. Risk factors for development of alpha-gal sensitization are of considerable interest particularly with regard to the highly tick-exposed groups like hunters and forest service employees compared to less frequently exposed subjects; e.g., borreliosis patients. Infections with the spirochete *Borrelia burgdorferi* are very common in tick-endemic areas like Southern Germany. Since ~ 10 – 20% of ticks in the state of Bavaria are infected with *Borrelia* and can transmit them seasonally, borreliosis can be a marker of previous tick contact. The tick must be attached for at least 36 – 48 hours before *Borrelia* can be transmitted to the host [11]. 

The aim of this study was to investigate the hypothesis that repeated tick exposure is a prominent risk factor for alpha-gal sensitization, explaining geographical and age-related differences. Are repeated exposures more important for sensitization than prolonged single exposures in line with a *Borrelia* infection? We compared the prevalence of IgE antibodies to alpha-gal in tick-exposed patients with borreliosis (with at least 1 tick bite of long duration) in comparison to frequently tick-exposed hunters and forest workers and the corresponding control group in Bavaria, Germany. Furthermore, we investigated whether elevated total IgE as an indicator for atopy is associated with sIgE to alpha-gal. Demographic risk factors were also analyzed. 

## Materials and methods 

### Study groups 

A total of 917 subjects, including 485 borreliosis patients (m = 192, f = 293) with different Lyme disease manifestations were enrolled in this study and compared to highly tick-exposed hunters and forest service employees (n = 232) as well as to a randomly selected control group from the same area (n = 200). The study was approved by the Ethics commission of the Medical Faculty of the Technical University of Munich. 

Blood sera of *Borrelia*-infected patients were grouped according to the clinical and serological manifestations of borreliosis in 343 patients with acute manifestations – early localized infection stage or erythema migrans (EM), furthermore 22 sera with multiple erythema migrans (MEM) as well as 120 patients with chronic borreliosis manifestation or the late disseminated stage – acrodermatitis chronica atrophicans (ACA) (Table 1). This study group consisted of 39.8% male and 60.2% female participants with a median age of 56 years. The corresponding blood sera were collected between 1995 and 2014 with an informed consent from all subjects and stored in the serum pool of the University Dermatology Department. 

Controls: A total of 200 randomly selected patients (m = 95, f = 105) were recruited after in- or outpatient treatment for different dermatological medical conditions like psoriasis, acne vulgaris, bullous pemphigoid, skin tumors (most commonly basal cell carcinoma), systemic lupus erythematosus, herpes zoster, eczema, erysipelas, leg ulcer, etc. in the University Department of Dermatology and Allergy of the Technical University, Munich. The age of these control patients varied between 12 and 91 years with a median age of 56, with 96 (48%) male and 104 (52%) female subjects. None of the controls were under suspicion for a Lyme borreliosis or received borreliosis treatment. 

The third survey population, at high risk for frequent tick exposure, was obtained by investigating hunters and forest service employees aged between 18 and 65 years. 56 sera from male hunters and forest workers, collected in 1988 around Munich in Bavaria, were stored in the University serum pool. Additionally, 176 new samples from hunters and members of hunting societies were obtained during the Trade Fair of hunters and hunting associations in Augsburg, Bavaria, in January 2016. The latter subjects reported a high number of tick bites during a 12-months period and had hunted for many years. 

Informed consent was obtained from each patient according to the guidelines of the Ethics commission; patients data were treated confidentially and anonymously. 

### Inclusion and exclusion criteria 

Inclusion criteria of the borreliosis group were previous tick bites confirmed by clinical and symptomatic borreliosis (acute or chronic disease manifestations as EM and other associated symptoms). *Borrelia* infections were serologically confirmed with at least one increased specific antibody response (IgG and/or IgM to *Borrelia burgdorferi*), determined by *Borrelia*-specific Immunoblot/ELISA (> 5 U/mL). To be enrolled, patients required at least one serological follow-up. Exclusion criteria of the borreliosis group were absence of a serological confirmation of *Borrelia* contact (IgG or IgM < 5 U/mL) and missing at least one follow-up visit in the Dermatology Department. Serologically positive patients with a positive *Treponema pallidum* test were also excluded (false positive result due to cross-reaction). 

The control population was randomly selected when admitted to in- or outpatient treatment in the University Department of Dermatology and Allergy without increased tick exposure, denying current borreliosis. Atopic patients were not excluded to avoid bias to the results of the genuine sample. Exclusion criteria were a current suspicion of *Borrelia* infection (including diagnosis and treatment). 

Inclusion criteria of the hunters and forest workers were either employment as a hunter or forest service employee, or a membership in a hunting society with longstanding hunting experience. Each individual was considered highly exposed to tick bites. 

### Sample processing and statistics 

Specific antibody assay ImmunoCAP 250 (Phadia AB/Thermo Fisher Scientific, Uppsala, Sweden) was used to measure the allergen-specific IgE antibodies against alpha-gal as well as to evaluate the total IgE levels in order to assess a possible atopy of all included subjects. All specimens were stored at 2 – 8 °C, or at minus 80 °C in the serum pool. In order to analyze the frequency of alpha-gal sensitization in different groups with tick exposure, we measured the positive IgE antibodies to alpha-gal using the ImmunoCAP assay with a cutoff of > 0.1 kU/L to detect low sensitization levels. sIgE below 0.1 kU/L was considered negative. 

Statistical analyses were performed in cooperation with the Institute of Medical Statistics of the Technical University of Munich. Continuous numerical variables were calculated as medians and interquartile ranges and compared by non-parametric Mann-Whitney test for independent variables. The Kruskal-Wallis test was applied to compare continuous numerical variables between the groups. Wilcoxon rank sum test and the two-sided nonparametric Spearman rho test for continuous and ordinal variables were used to assess correlations or to obtain a correlation coefficient, respectively. The relative risks for comparison of the three different groups were calculated from contingency tables. 

A p-value of < 0.05 was considered statistically significant. For the statistical acquisition of the data and their interpretation, the computer programs Microsoft Excel, Matlab, and SPSS Statistics (IBM SPSS Statistics 25) were used. 

## Results 

### Alpha-gal sensitization rates, risk, and exposure 

**Figure 1:**
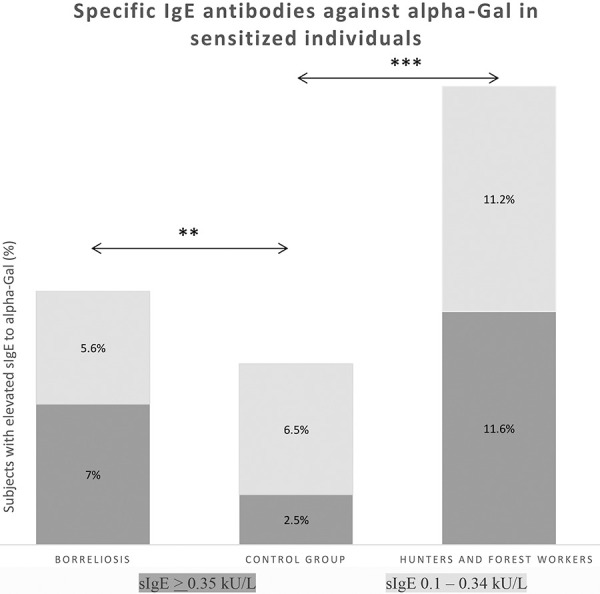
Prevalence of elevated specific IgE antibodies to alpha-gal in the tested populations. The prevalence of specific IgE to alpha-gal was significantly higher in hunters/forest workers than in borreliosis patients and controls. **p < 0.01, ***p < 0.001. Continuous numerical variables were calculated as medians and interquartile ranges and compared by the non-parametric Mann-Whitney test for independent variables. The Kruskal-Wallis test was applied to compare continuous numerical variables between the groups.

The prevalence of alpha-gal sIgE in the two tick exposed groups and the control population is presented in Table 1. The table shows sIgE antibodies grouped according to acute and chronic borreliosis manifestations and sIgE titers. Elevated sIgE to alpha-gal among borreliosis patients was demonstrated in 12.6% with > 0.1 kU/L. In contrast, the evaluation of the tick-exposed hunters and forest workers revealed as many as 22.8% sensitized individuals. Specific sensitization was significantly lower (9%) in the control population (Table 1) (Figure 1). Forest workers from 1988 and from 2016 showed a very similar rate of elevated sIgE to alpha-gal (21.4% and 23.3%, respectively). 

*Borrelia*-associated alpha-gal IgE sensitization showed a median level of 0.46 kU/L (range 0.10 – 11.7 kU/L). The rate of elevated IgE antibodies varied between 11.5 and 15.8% in the different groups with manifestations of borreliosis. These manifestations as a measure of chronicity were not correlated to sensitizations. Hunters presented median levels of 0.35 kU/L (range 0.1 – > 100 kU/L) in comparison to the controls with a lower median level of 0.21 kU/L (range 0.1 – 1.22 kU/L). Most of the individual titers were generally low, rarely exceeding 3.5 kU/L. Two samples in the forest service employee group showed IgE antibodies against alpha-gal >50 kU/L. 

A statistically significant difference in sIgE positivity can be demonstrated comparing both tick-exposed groups. Significant differences were also shown comparing the borreliosis and control group (p = 0.008) and between hunters and the control group (p < 0.001) (Figure 1). Moreover, a statistically significant difference between forest workers and borreliosis patients could be demonstrated with regard to the sIgE levels (p < 0.001, elevated in both groups), although there is a different frequency in sensitization. 

For the two tick-bite exposed groups, a different increased relative risk for developing alpha-gal sensitization versus controls is summarized in Table 2. Hunters and forest service employees show a significantly higher relative risk for alpha-gal sensitization of 2.5 (95% CI 1.5 – 4.2; p < 0.001) compared to 1.4 in tick-exposed borreliosis patients (95% CI 0.85 – 2.3; not significant (n.s.)). 

### Demographic risk factors 

**Figure 2:**
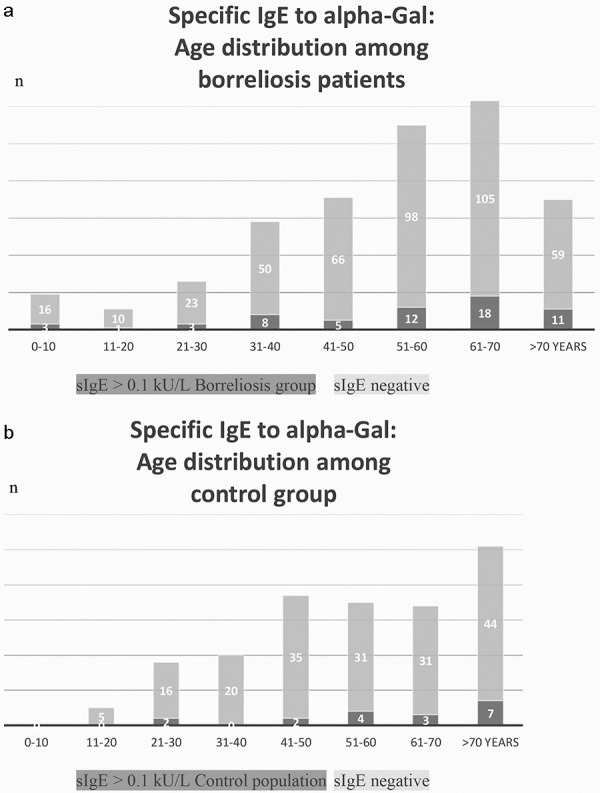
Age distribution among borreliosis patients (a) and control group (b). Specific IgE to alpha-gal > 0.1 kU/L, absolute numbers. An increased sensitization in patients older than 50 years was observed in both groups.

The distribution of age among the borreliosis group in relation to IgE alpha-gal status is shown in Figure 2. Higher sensitization frequencies occur in patients at the age of 50 and older. Similarly, increased alpha-gal IgE was noted more often in the controls of ≥ 50 years. 14/18 sensitized control patients were aged over 50 years, including 7 individuals over 70 years old. 

Comparing gender in the borreliosis subgroups, a total of 42 patients with IgE antibodies to alpha-gal can be seen in the acute manifestation form, EM and MEM (17 female and 25 male subjects), respectively 40% and 60% of all sensitized in early borreliosis manifestation. Of all EM patients this means 7% male and 3.5% female patients showed alpha-gal specific IgE. In patients with the late chronic borreliosis manifestation (ACA), 12 females and 7 male (respectively 10% and 6% of all ACA) individuals were tested positive for specific IgE antibodies against alpha-gal compared to 9 male and 9 female patients (4.5% each gender) tested positive in the control group. 

### Total IgE 

An association of elevated total IgE (> 100 IU/mL, designating atopic patients) and specific IgE to alpha-gal could be observed in this study. 

Atopy ranged from 19.6% (n = 95/485) in the borreliosis group, 29.5% (n = 59/200) in controls up to 31.5% (n = 73/232) in hunters and forest service employees. A statistically significant difference could be demonstrated between the groups (p < 0.001). 

Alpha-gal sensitization was observed more frequently in atopic individuals compared to non-atopic ones. 30.8% (n = 70/227) of all atopic subjects showed elevated alpha-gal sIgE in contrast to 9% (n = 62/690) of non-atopic subjects in the differently tick-exposed groups. Statistically significant differences were shown between alpha-gal-sensitized and alpha-gal-unsensitized patients, since alpha-gal-sensitized subjects showed higher total IgE antibodies (mean rank 650.9 vs. 426.7 IU/mL). 

## Discussion 

Due to the mutation of the alpha-1,3-galactosyltransferase gene in humans and Old World Monkeys over 20 million years ago, alpha-gal became immunogenic to humans allowing the production of anti-alpha-gal antibodies, usually of the IgG type [20, 21]. Factors contributing to sIgE formation are of higher interest because there are patients with alpha-gal syndrome. 

Previous work suggested a correlation between tick contact and an IgE-mediated sensitization to alpha-gal. In this study we showed that alpha-gal sensitization is not significantly increased in patients with borreliosis (relative risk = 1.4 vs. controls; 95% CI 0.85 – 2.3) in whom we suspect only a singular or rare tick contacts. Interestingly, highly tick-exposed individuals like hunters and forest service employees show higher and even more frequently elevated alpha-gal IgE antibodies (relative risk = 2.5; 95% CI 1.5 – 4.2). Therefore, frequent tick contact seems to be more important than a single or rare (even prolonged) tick attachment of several hours in order to acquire sensitization. *Borrelia*-specific IgG and IgM decrease over time and are not in correlation with specific alpha-gal IgE. Taken together, results suggest a relevant association between tick exposure and alpha-gal sensitization: the more frequent the tick contacts are, the more likely is sensitization. We assume that in many patients, a single tick contact, as frequently found in patients with borreliosis, is not sufficient to induce permanent sIgE production and an alpha-gal sensitization. The relatively low antibody titers in alpha-gal-positive subjects are in line with previous findings of other studies [1, 2, 3, 17, 19]. Fischer et al. [1] could demonstrate that 8.6% of sensitized forest workers (> 0.35 kU/L) became symptomatic following alpha-gal-containing meat exposure. The same study demonstrated sIgE to decrease over time after avoiding additional tick bites, indicating that also allergic symptoms (urticaria, angioedema, and extracutaneous manifestations like nausea, vomiting, anaphylaxis) may disappear in the affected individuals when re-exposure to ticks can be avoided. Commins et al. [22] earlier showed that allergic patients tolerated mammalian meat after 1 – 2 years of avoidance following tick bites. 

Recent studies found atopy to be a predisposing factor to develop severe alpha-gal allergy [23]. In accordance with previous studies, we found that elevated total IgE levels as a covariate of atopy are associated with increased sIgE to alpha-gal: we demonstrate a higher rate of elevated total IgE in alpha-gal-sensitized individuals, especially in hunters and forest workers (41.1%). Compared to the controls (18.6% elevated total IgE), both tick-exposed groups showed significantly higher total IgE and sIgE to alpha-gal. An increase of alpha-gal positivity by 2% per 10 kU/L total IgE in atopic individuals has been demonstrated by Fischer et al. [1]. We confirm these data on the importance of atopy as a predisposing factor for the development of alpha-gal sensitization. 

In general agreement with the results of previous studies, we found elevated alpha-gal IgE in patients aged 50 years and older. This sensitization late in life may be explained by a cumulative (booster) component which increased over time and outweighed the phenomenon of immunosenescence in the elderly (higher risk to develop alpha-gal sensitization due to the accumulated number of tick bites in the course of a lifetime). Cofactors like physical activity or alcohol are able to trigger an allergic response in sensitized patients due to the increased intestinal absorption and could also be identified in 81% of symptomatic patients after oral exposition to alpha-gal [29]. In contrast to previous studies [24], we found no association between alpha-gal sensitization and male gender. As described, male and female individuals were distributed nearly equally without a male predominance and without a significant difference in gender in our balanced population (borreliosis and control group, lack of data in hunters). A similar finding was also demonstrated in a Spanish cohort, but not in other recently studied groups in which a male predominance in sensitized individuals was detected. It is not clear whether the difference in gender distributions is related to different outdoor activities including the frequency, difference in clothing, or even different tick removal strategies. Further investigations need to assess the gender distribution in risk groups among alpha-gal-sensitized patients. 

In our study, IgE sensitization to alpha-gal was found in 12.6% of borreliosis patients, in 22.8% of hunters, and in 9% of controls. Alpha-gal IgE prevalence was within the range (5.5 – 24.5%) that has been found in the United States and different European countries. However, alpha-gal sensitization in the subgroup in the area of Munich is lower than in the state of Baden-Württemberg (35% in forest service employees) and lower than in the United States which may be due to different tick species (USA) or due to a different frequency of tick contact or tick removal strategies. Approximately 10 – 20% of ticks in Bavaria carry *Borrelia burgdorferi*. In this sense, it is important to keep in mind asymptomatic tick bites without a subsequent borreliosis infection that may remain undetected and still induce a sensitization to alpha-gal. Beyond the scope of this study, we propose to compare the variation in tick abundance over different regions (in Germany) and to find out the difference between the state of Bavaria as a tick-endemic area in contrast to the state of Baden-Württemberg as the closest region. Villalta et al. [17] previously described differences between rural and urban areas in this regard. Another important explanation for different results in sIgE and borreliosis association [24] may be represented by the sampling time, as tick populations are highest between April and October and decrease over the winter. Favorable seasonal conditions in years with a high tick population hereby increase the prevalence of alpha-gal sensitization by multiple tick bites in risk groups. Moreover, the tick population increases with the deer and rodent population as previously reported from Sweden and the USA, and blood type may also be a confounder [25, 26, 27, 28]. 

The fact that there were sensitized forest workers in 1988 may explain many unsolved cases of idiopathic anaphylaxis in earlier years. However, individual allergy data on this study group were not available, due to data protection reasons. This study could show that alpha-gal sensitization and presumably alpha-gal syndrome does not seem to be a modern problem but existed already 30 years ago. Further work should focus on symptomatic patients in a sensitized cohort. It may be beneficial to establish IgE screening for risk populations in order to detect a possible alpha-gal sensitization before the first symptoms can occur, as titers may decrease with re-exposure avoidance. A prospective study may define recommendations, e.g., for hunters, concerning the behavior after tick bites. 

## Conclusion 

In conclusion, this study emphasizes that frequent tick exposure leads to alpha-gal sensitization. Subjects with more tick exposure, like forest workers and hunters, represent a population at risk for the development of alpha-gal sensitization: these groups have more than twice the relative risk to acquire alpha-gal sensitization. The presumably less frequently tick-exposed borreliosis cohort still shows one and a half the relative risk in comparison to the control cohort (n.s.). Therefore, sensitization to alpha-gal cannot be easily acquired in a population with low/minor tick exposure. Antibody response to alpha-gal seems to be more frequent in older and atopic subjects. Questions remain about the nature of the sensitization process and the development of the full alpha-gal syndrome. 

## Acknowledgment 

We thank Phadia Thermo Fisher for providing material for alpha-gal antibody assays. 

## Authors’ contributions 

MB substantially contributed to the acquisition, analysis, and interpretation of data, discussion, and writing. UD contributed to study design and planning, evaluation, discussion, and writing of the manuscript. MK provided access to patients’ data/database, laboratory data, computing resources, and contributed to data collection. HH provided access to laboratory data/*Borrelia* database and computing resources. TB conceptualized the project, formulated research goals and aims, substantially contributed to interpretation of data, revised critically for important intellectual content, and reviewed and approved the final version of the manuscript. All authors provided critical revision of the manuscript for important intellectual content. They all gave their agreement for the publication. 

## Funding 

Thermo Fisher Scientific provided laboratory material for this study. 

## Conflict of interest 

TB was investigator for Thermo Fisher Scientific and reports non-financial support (laboratory material and analytical support) from Thermo Fisher Scientific. All other authors report no conflict of interest in relation to this work. 

**Table 1.** Borreliosis stage and elevated specific IgE antibodies against alpha-gal with a threshold of > 0.1 kU/L; population at high risk for tick bites divided into forest workers from 1988 and forest service employees, hunters, and members of hunting societies, and patients (acute borreliosis: erythema migrans; chronic borreliosis: acrodermatitis chronica atrophicans).

**Population**	**Number of patients**	**0,1 – 0.69 ** **CAP 0 – 1 ** **n**	**0.70 – 3.49 CAP 2 ** **n**	**3.50 – 17.49 ** **CAP 3 ** **n**	**17,50 – 49,99 ** **CAP 4 ** **n**	**50.0 – 99.99 ** **CAP 5 ** **n**	**> 100 kU/L ** **CAP 6 ** **n**	**n ** **(% of total)**
Acute borreliosis	365	28	12	2	0	0	0	42 (11.5%)
Chronic borreliosis	120	13	2	4	0	0	0	19 (15.8%)
								Total: 61 (12.6%)
Forest workers 1988	56	5	3	3	0	0	1	12 (21.4%)
Forest service employees 2016	176	23	8	7	2	1	0	41 (23.3%)
								Total 53 (22.8%)
Control group	200	16	2	0	0	0	0	Total 18 (9%)

sIgE = specific IgE.

**Table 2.** Comparison of the relative sensitization risk in two tick-exposed populations. Tick bite-exposed groups show different increased relative risk for developing alpha-gal sensitization compared to controls.

	**RR**	**95% CI**	**p**
Borreliosis group	1.4	0.85 – 2.3	(n.s.)
Hunters	2.5	1.5 – 4.2	p < 0.001

RR = relative risk; 95% CI = 95% confidence interval; n.s. = not significant.
